# Placental volume as a novel sign for identifying placenta accreta spectrum in pregnancies with complete placenta previa

**DOI:** 10.1186/s12884-024-06247-y

**Published:** 2024-01-10

**Authors:** Yongfei Yue, Xiaoyan Wang, Liping Zhu, Chengfeng Liu, Dali Chen, Yanli Lu, Baoquan Liang

**Affiliations:** 1 Department of Obstetrics and Gynecology, The Affiliated Suzhou Hospital of Nanjing Medical University, Suzhou Municipal Hospital, No. 26 Daoqian Street, Gusu District, Suzhou, 215002 Jiangsu China; 2 Department of Medical Imaging, The Affiliated Suzhou Hospital of Nanjing Medical University, Suzhou Municipal Hospital, Suzhou, Jiangsu China

**Keywords:** Complete placenta previa, Cesarean section, Magnetic resonance imaging, Placenta accreta spectrum, Placental volume

## Abstract

**Background:**

Placenta accreta spectrum (PAS) carries an increased risk of maternal-fetal mortality and morbidity, and magnetic resonance imaging (MRI) features for PAS have been used for preoperative identification. This study aims to investigate the role of placental volume evaluated by MRI in identifying PAS in pregnant women with complete placenta previa.

**Methods:**

Totally 163 cases of complete placenta previa pregnant women with a history of cesarean section underwent MRI for suspected PAS were included. We categorized the patients into two groups according to the presence or absence of PAS, and the maternal-fetal perinatal outcomes and placental volume analyzed by 3D Slice software were compared.

**Results:**

There were significantly more gravidity, parity, and number of previous cesarean delivery in the PAS group (*P* < 0.05). Significant differences were also found between the two groups with respect to the following baseline characteristics: gestational age at delivery, intraoperative blood loss, blood transfusion, and neonatal birth weight (*P* < 0.05). Of 163 women in the study, 7 (4.294%) required cesarean hysterectomy for high-grade PAS or pernicious bleeding during cesarean section, and PAS was confirmed with histologic confirmation in 6 (85.714%) cases. The placental volume in PAS group was greater than that in the non-PAS group (*P* < 0.05). With a threshold of more than 887 cm^3^, the sensitivity and specificity in identifying PAS were 85.531% and 83.907% respectively, with AUC 0.908 (95% CI: 0.853–0.948).

**Conclusions:**

Placental volume may be a promising indicator of PAS in complete placenta previa patients with a history of cesarean section.

## Introduction

 Placenta accreta spectrum (PAS) is defined as abnormal attachment and adherence of the placenta to the uterus, which may result in severe maternal morbidity or death [1]. Over the past decades, with the increase of cesarean section rate, the incidence of PAS was also rising [2]. As a previous report described, cesarean deliveries were independent clinical risk factors of PAS [3]. Placenta previa is the highest risk factor for PAS, with an odds ratio (OR) value of 50.75 in these patients.[4] Furthermore, placenta previa with PAS can lead to higher maternal-fetal mortality and morbidity (premature delivery, massive hemorrhage, urinary tract injury and hysterectomy, etc.) than those without PAS [5, 6].

Antenatal diagnosis and assessment of the severity of the disease are critical to optimize delivery management, which could reduce mortality and morbidity. Currently, ultrasound is the preferred test for detecting PAS due to its low cost and high accuracy [7]. However, for posterior placenta, obese pregnant women and pregnant women with gastrointestinal pneumatosis, the advantages of magnetic resonance imaging (MRI) are more obvious [8]. The useful MRI features for PAS include placental thickness, dark intraplacental band on T2-weighted images (T2WIs), placenta-myometrial interface, placental heterogeneity, abnormal intraplacental vascularity, uterinebulging and so on [9, 10]. It also has been shown that the placental area have important impact on the clinical outcomes of patients with complete placenta previa [11]. However, placental volume of PAS is less studied. Moreover, more accurate MRI findings for predicting complete placenta previa with PAS are needed. In this context, this study aimed to investigate the relationship between placental volume and the likelihood of PAS in pregnancies with complete placenta previa with a history of cesarean section.

## Materials and methods

### Study Population and MRI Protocol

 This study was approved by our institutional Ethics Review Board (Approval K-2022-015-K01). Informed consent was waived because of the retrospective nature of this study with anonymous selection, which did not subject the patients to new interventions. We reviewed the clinical data of complete placenta previa pregnant women with a history of cesarean section between January 2016 and December 2022. The inclusion criteria were as follows: (1) > 18 year old pregnant patients with complete placenta previa detected by ultrasound, (2) patients with suspected PAS disorders detected by both ultrasound and MRI in the third trimester, (3) patients with singleton pregnancy confirmed by early ultrasound before 15 weeks gestation, (4) with a history of more than one cesarean section. Exclusion criteria: (1) patients with twin or multiple pregnancies, (2) Patients conceived by assisted reproductive technology, (3) patients for whom MRI images were not retrieved on incomplete for evaluation, (4) poor image quality (motion artifacts, increased noise, wraparound artifacts, etc.).

Patients histories and intraoperative information was obtained from the electronic medical record system. The diagnosis of complete placenta previa was based on transabdominal ultrasound finding that the placental tissue completely covered the internal cervical os, and further MRI was performed if PAS was highly suspected. The included patients were classified into PAS and non-PAS groups based on FIGO criteria. Both clinical and histologic FIGO criteria were used to classify the grade of PAS, including grade 1 for accreta, grade 2 for increta, and grade 3 (3a, 3b and 3c) for percreta [12]. A pathologic diagnosis of PAS was identified on a review of hysterectomy specimens.

The MRI examinations without gadolinium were performed on 3 T (Siemens Medical Solutions, Erlangen, Germany) ranged from 31 to 34 gestational weeks. The pregnant women were asked to keep the urinary bladder moderately distended in order to evaluate the bladder-serosal interface during the MRI examination. Most patients were examined in the supine position and a small number of patients who could not tolerate this position examined in the left lateral decubitus position. MRI images were acquired using T_2_-weighted half-fourier acquisition single shot turbo spin echo (HASTE) sequence (repetition time 700 ms, echo time 87 ms, bandwidth 698 Hz/px, 432 × 432 matrix over a field of view of 380 × 380 mm, 5 mm slice thickness). The axial, sagittal and coronal planes were included with respect to the uterus.

**Fig. 1:**
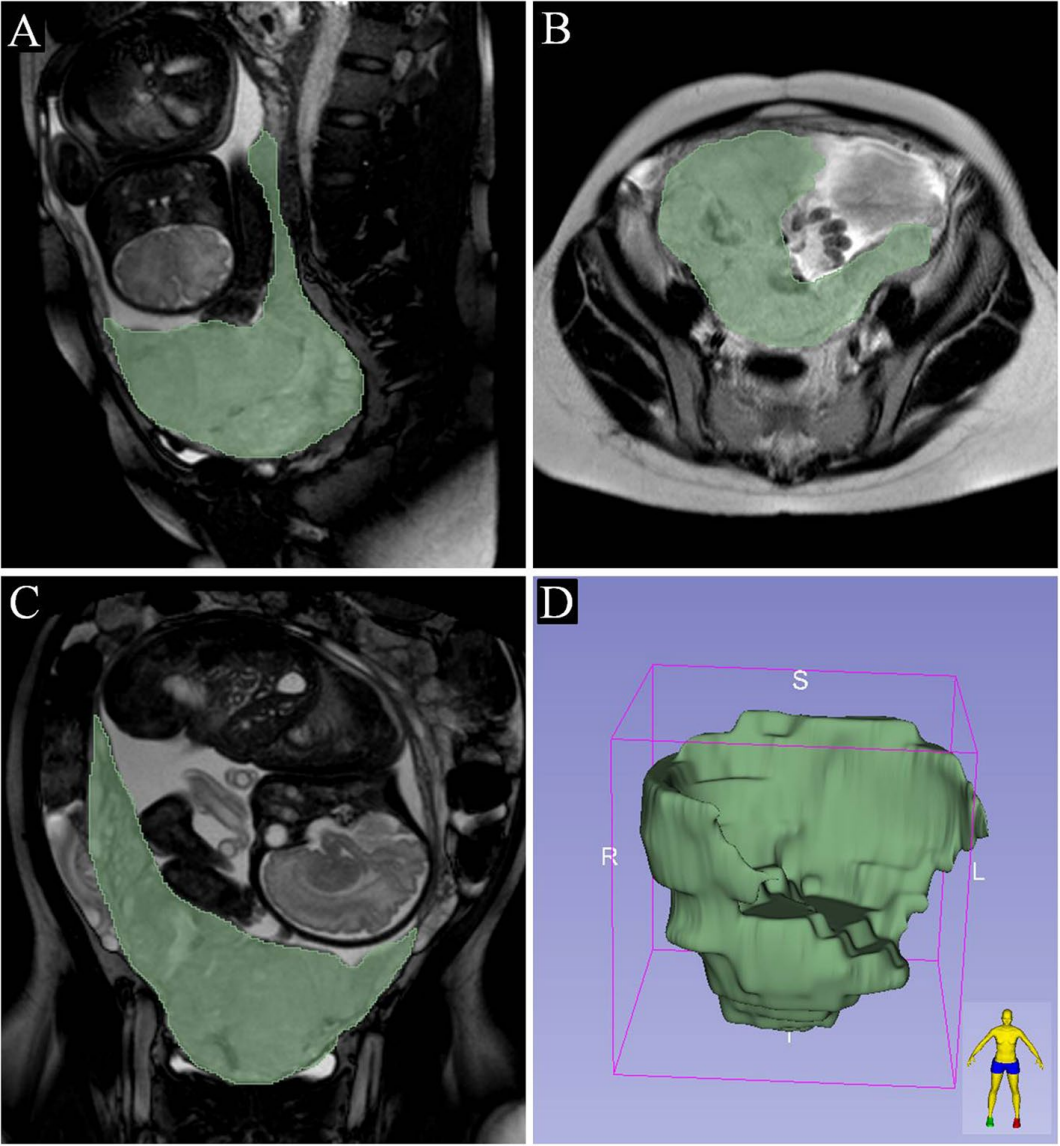
Placental volume was reconstructed in patients with complete placenta previa by 3D slicer software. **A** Sagittal MRI image with green color overlay from manual segmentation. **B** Axial MRI image with green color overlay from manual segmentation. **C** Coronal MRI image with green color overlay from manual segmentation. **D** Three-dimensional  placenta image generated by 3D Slicer using each manually segmented slice

The placental volume was independently measured by 2 radiologists specializing in obstetric and gynecologic imaging diagnosis (with more than 20 years of experience, respectively). The radiologists evaluated MRI images without knowledge of the clinical data of each patient. Interobserver measurements were compared for variability (kappa). The MRI images of patients with complete placenta previa were imported into 3D Slicer software (version 5. 2.1, www.slicer.org) in order to create placental profile and measure placental volume. The main steps were as follows: (1) import the patient’s original MRI images in DICOM format, (2) run the Editor module under the 2-dimensional window, (3) performe segmentations to trace the outer contours of the placenta manually on each slide, (4) use the 3-D segmentation function of the program, the placental volume of all included voxels were calculated (Fig. 1).

### Statistical analysis

Continuous variables were expressed as mean ± standard deviation (SD) and categorical variables were summarised using counts and proportions. The statistical analysis included the t-Test, Chi-square, and receiver operating characteristic (ROC) curves. The ROC curve was analyzed to evaluate the area under the curve (AUC). Based on the ROC curve, the appropriate cut-off value set of placental volume, sensitivity, specificity, positive predictive value (PPV), and negative predictive value (NPV) were calculated. The agreement between the two measurements was checked by kappa scores according to the following definitions: 1.0 perfect agreement, 0.91 to 0.99 almost perfect agreement, 0.81 to 0.90 substantial agreement, 0.71 to 0.80 moderate agreement, 0.61 to 0.70 fair agreement, and < 0.6 slight agreement. *P* value less than 0.05 were considered statistically significant. All statistical analyses were carried out using SPSS 23.0 statistics software (version 23.0, SPSS Inc., Chicago, IL, USA).

## Results

In the current study, 163 women with at least 1 prior cesarean section received transabdominal ultrasound and MRI diagnosis of complete placenta previa and delivered at our institution. Of which 76 had confirmed PAS and 87 had simple complete placenta previa. Table 1 shows the clinical characteristics of these patients. There were significantly more gravidity, parity, and number of previous cesarean delivery in the PAS group (*P* < 0.05). Significant differences were also found between the PAS and non-PAS groups with respect to the following baseline characteristics: gestational age at delivery and neonatal birth weight. Women who had PAS had higher intraoperative blood loss (2502.829 ± 718.001 mL vs. 1951.713 ± 685.262 mL) and required more blood product transfusion (1940.487 ± 441.443 mL vs. 1353.103 ± 582.038 mL) compared to those without PAS (*P* < 0.001). Of 163 women in the study cohort, 7 (4.294%) required cesarean hysterectomy for high-grade PAS or pernicious bleeding during cesarean section, and PAS was confirmed with histologic confirmation in 6 (85.714%) cases. However, there were no statistical significances in terms of maternal age, BMI, previous history of placental previa, and gestational age at screening by MRI between the two groups.

**Table 1:** Patients clinical features of the study groups

Parameter	Patients with PAS (*n* = 76)	Patients without PAS (*n* = 87)	Statistic	* P* value
Maternal age	31.895 ± 4.012	30.828 ± 3.597	t = 1.791	0.075
BMI (kg/m^2^)	25.659 ± 3.249	26.051 ± 4.239	t = 0.658	0.512
Gravidity			7.219	0.007
2	15 (19.737)	34 (39.080)		
> 2	61 (80.263)	53 (60.920)		
Parity			6.809	0.009
2	43 (56.579)	66 (75.862)		
> 2	33 (43.421)	21 (24.138)		
Number of previous cesarean delivery			10.200	0.001
1	53 (69.737)	78 (89.655)		
> 1	23 (30.263)	9 (10.345)		
Previous history of placental previa	6 (7.895)	2 (2.299)	2.722	0.099
Gestational age at screening by MRI (weeks)	32.880 ± 0.733	33.029 ± 0.896	t = 1.147	0.253
Gestational age at delivery (weeks)	35.349 ± 1.370	36.000 ± 1.129	t = 3.327	0.001
Neonatal birth weight (g)	2474.474 ± 434.957	2687.356 ± 468.249	t = 2.993	0.003
Intraoperative blood loss (mL)	2502.829 ± 718.001	1951.713 ± 685.262	t = 5.001	< 0.001
Blood transfusion (mL)	1940.487 ± 441.443	1353.103 ± 582.038	7.177	< 0.001
Hysterectomy	6 (7.895)	1 (1.149)	4.491	0.034

*Abbreviations*: *MRI *Magnetic resonance imaging

**Fig. 2:**
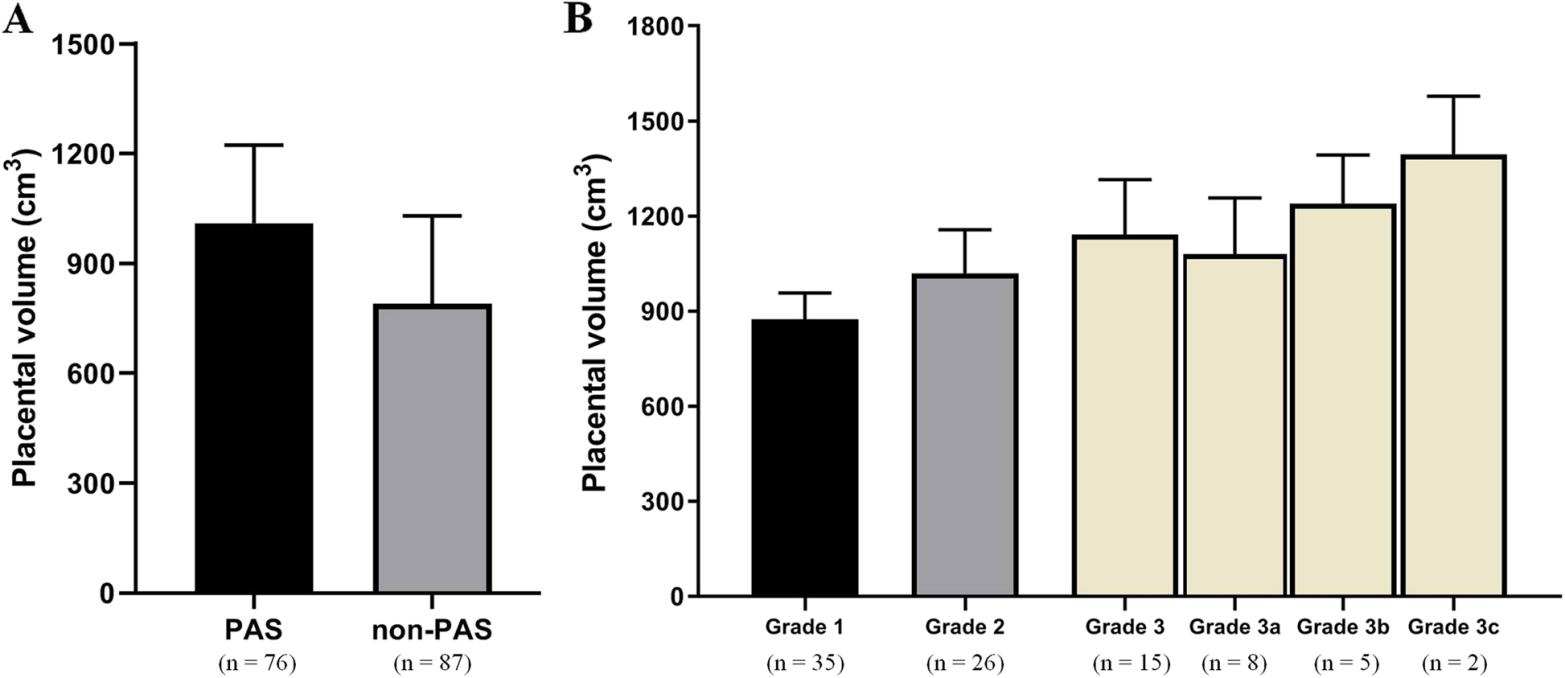
**A**. Placental volume between placenta accreta spectrum (PAS) and non-placenta accreta spectrum (non-PAS) group. **B** Placental volume in PAS patients with different grades by FIGO criteria

**Fig. 3:**
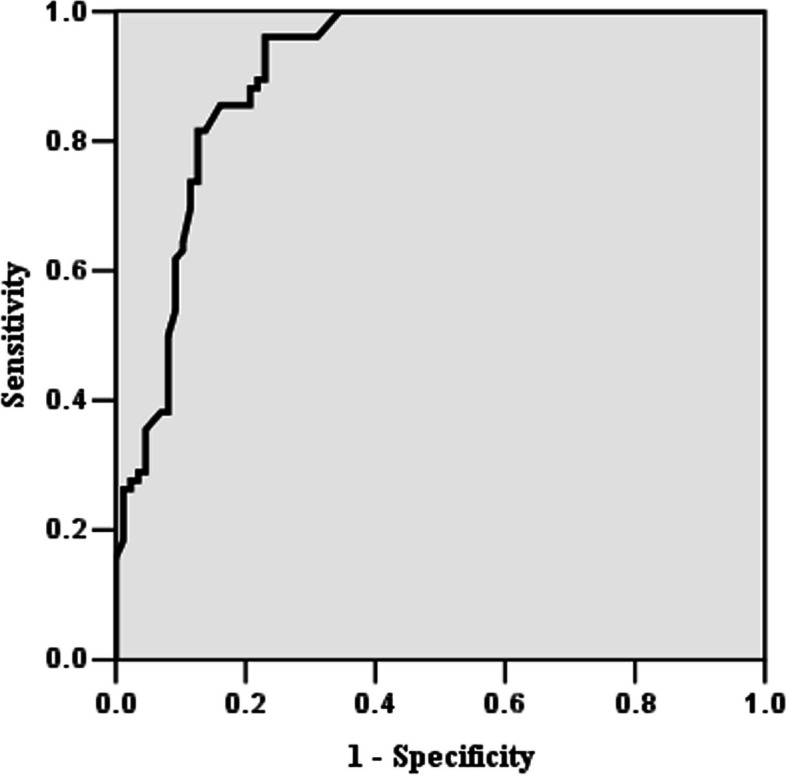
Receiver operating characteristic (ROC) curve showing the predictive performance of placental volume for the occurrence of placenta accreta spectrum (PAS) in women with complete placenta previa with a history of cesarean section. With a threshold of more than 887cm^3^, the area under the curve (AUC) was 0.908 (95% CI: 0.853-0.948)

Here, 3D Slicer software was used to perform three-dimensional reconstruction and calculate the volume of placental volume (Figs. 1 and 2). There was good agreement for the measurement of placenta volume between the two radiologists (kappa = 0.938) (Table 2). The placental volume in PAS group was greater than that in the non-PAS group (*P* < 0.05) (Fig. 2A). According to clinical and histologic FIGO criteria of PAS, the depth of invasion was accreta or grade 1 in 35 (46.053%), increta or grade 2 in 26 (34.211%), and percreta or grade 3 in 15 (19.737%). As the grade increased in PAS patients according to FIGO criteria, the placental volume also increased(Fig. 2B). The sensitivity, specificity, PPV, and NPV of each placental volume cut-off value for the prediction of PAS were calculated and shown in Table 3. With a threshold of more than 887 cm^3^, the sensitivity and specificity in identifying PAS were 85.531% and 83.907% respectively, with AUC 0.908 (95% CI: 0.853–0.948) (Fig. 3).

**Table 2:** Interobserver reliability of magnetic resonance imaging (MRI) in the measurement of placental volume

MRI features	Either	All	Agree	Kappa	Interpertation
Placental volume	77(47.239)	72(44.172)	96.933	0.938	Almost perfect

**Table 3:** Cut-off levels of placental volume for the occurrence of placenta accreta spectrum (PAS).

	Cut-off	Sensitivity % (95% CI)	Specificity % (95% CI)	PPV %	NVP %	P
Placental volume (cm^3^)	760	97.373 (90.783–99.625)	67.824 (56.876–77.365)	72.548	96.680	< 0.001
Placental volume (cm^3^)	790	96.053 (88.873–99.148)	77.014 (66.769–85.353)	78.530	95.738	< 0.001
Placental volume (cm^3^)	840	89.472 (80.284–95.348)	78.163 (68.046–86.327)	78.187	89.560	< 0.001
Placental volume (cm^3^)	860	88.153 (78.707–94.364)	79.31 (69.307–87.172)	78.840	88.485	< 0.001
Placental volume (cm^3^)	887	85.531 (75.563–92.455)	83.907 (74.547–90.906)	82.273	86.906	< 0.001
Placental volume (cm^3^)	896	81.58 (71.037–89.540)	87.36 (78.485–93.548)	84.936	84.437	< 0.001
Placental volume (cm^3^)	935	73.683 (62.263–83.061)	88.51 (79.949–94.339)	84.806	79.384	< 0.001
Placental volume (cm^3^)	980	64.473 (52.674–75.109)	89.663 (81.273–95.138)	84.543	74.263	< 0.001

*Abbreviations*: *PPV *Positive predictive value, *NPV N*egative predictive value

## Discussion

PAS in complete placenta previa is a life-threatening obstetric complication, which can cause massive antepartum or postpartum hemorrhage for mothers. With the growth rate of cesarean deliveries, the incidence of PAS is also gradually increasing, prenatal diagnosis of PAS is vital. Accurate prenatal diagnosis of PAS can give obstetricians enough time to develop a multidisciplinary approach to delivery planning, which can decrease maternal and neonatal morbidity, including massive maternal hemorrhage and intensive care unit hospitalizations [13]. Thus, predicting PAS with high sensitivity and specificity for complete placenta previa is significantly useful in clinical settings. Prenatal diagnosis of PAS requires comprehensive analysis of risk factors and imaging data of pregnant women. In accordance with the literature, there are plenty of MRI features have been considered useful for predicting PAS, such as dark T2 bands, short cervix, abnormal uterine bulging, [14, 15] and so on. The international society for abnormally invasive placenta (IS-AIP) proposed several standard or agreement of MRI signs for PAS, including heterogeneous placenta, placental bulge, dark intraplacental bands, placental ischemic infarction, loss of retroplacental dark zone, and so on [16]. These MRI signs proposed by IS-AIP were high-risk signals for PAS. This study identified PAS by measuring placental volume, which might provide a new idea for screening PAS before cesarean section. As far as we know, there are no previous reports exploring the association between placental volume and PAS. We introduce a novel method for the analysis of PAS by measuring placental volume based on MRI.

Placenta previa and previous cesarean section are the most important independent risk factors for PAS [17]. PAS patients with the combination of placenta previa and a prior caesarean delivery have a higher risk of postpartum hemorrhage and hysterectomy [18]. Complete placenta previa was an obstetric complication different from incomplete placenta previa and associated with morbidity and mortality of the mother and neonate [19]. Therefore, the study population included in this study was women with complete placenta previa and a history of cesarean delivery. The main cause of PAS is the endometrium and myometrium damage caused by cesarean section. The trophoblast and villi may invade the myometrium and even pelvic organs through the endometrial defect of the uterine scar [20]. Jung EJ et al. [21] found that patients with placenta previa have large chorionic plate diameters and chorionic plate areas than normal pregnant women. The pathogenesis of abnormal placental vascularization is still under debate. Several studies have reported that the abnormally rich vascularity between the uterine scar and the placenta was observed in the first trimester of PAS patients [8, 22]. In patients with complete placenta previa, the placenta is attached to the lower uterine segment with poorly blood supply, and the compensatory growth of placental villi and increased placental volume are conducive to increased placental perfusion. The proliferated placental villi may implant in the myometrium and even penetrate the serosa layer of the uterus, which can allow the placenta and fetus to receive more blood supply and nutrition. Therefore, it is speculated that abnormal invasion of trophoblast and rich vascularity are important reasons for the increase of placental volume in PAS patients.

Most authors believe that the differentiation of placenta adherenta, increta and placenta percreta by MRI is challenging. Definitive MRI screening of PAS require findings of obvious placental extrauterine spread [23]. The previously reported sensitivity and specificity of MRI in the screening of PAS ranged from 75 to 100% and from 65 to 100%, respectively [9]. Rezk et al. [24] used dark intraplacental bands of MRI to predict PAS with a sensitivity and specificity of 92.73% and 76.00%, and the overall sensitivity and specificity was 96.08%, 87.50% by combining with three MRI features, respectively. In this study, women with PAS have larger placental volume than those women without PAS. When the placental volume cut-off value was 887 cm^3^, it showed excellent sensitivity (85.531%) and specificity (83.907%) for the identification of PAS, with excellent interobserver agreement (kappa = 0.938). The screening of PAS in most previous studies required the presence of multiple MRI parameters examined. Although the sensitivity and specificity are not the highest in this study, we did rely solely on placental volume, which could be quantified by 3D Slicer software. A key finding of our study is that measurements of the placental volume could provide additional information to assist us in identifying PAS. To our knowledge, this study is the first to test this imaging feature on MRI. In this study, the cases with PAS have more intraoperative blood loss compared with the group without PAS, which might have induced the higher frequency of hysterectomy. There were a low rate of hysterectomy in this study, which is due to an interprofessional team in managing PAS patients. Adequate preoperative evaluation and skilled surgical operation (uterine artery ligation, anti-arcuate compression suturing, and B-lynch suturing, etc.) of our team can significantly improve outcomes of mother and fetus [25]. The low rate of hysterectomy made histopathology difficult to perform in this study. Therefore, it was very necessary to diagnosis and classify PAS by the combination of clinical and histopathological criteria [26].

## Limitations

The limitations of this study include the possibility of bias due to the retrospective nature of this study and the potential incidence of uterine dehiscence, which is considered in this study. Additional prospective studies are required to evaluate clinical and histopathological significance related with placental volume in predicting PAS.

## Conclusion

In conclusion, placental volume based on MRI can be used to evaluate whether PAS is present in patients with complete placenta previa with a history of cesarean section.

## Data Availability

All data generated or analysed during this study are included in this published article.

## Abbreviations

PAS: Placenta accreta spectrum
MRI: Magnetic resonance imaging
OR: Odds ratio
T2WIs: T2-weighted images
HASTE: Half-fourier acquisition single shot turbo spin echo
SD: Standard deviation
ROC: Receiver operating characteristic
AUC: Area under the curve
PPV: Positive predictive value
NPV: Negative predictive value
IS-AIP: International society for abnormally invasive placenta
